# Feasibility of using smartphones by village health workers for pregnancy registration and effectiveness of mobile phone text messages on reduction of homebirths in rural Uganda

**DOI:** 10.1371/journal.pone.0198653

**Published:** 2018-06-27

**Authors:** Gershim Asiki, Robert Newton, Leonard Kibirige, Anatoli Kamali, Lena Marions, Lars Smedman

**Affiliations:** 1 Department of Women’s and Children’s Health, Karolinska Institutet, Stockholm, Sweden; 2 Medical Research Council/Uganda Virus Research Council, Uganda Research Unit on AIDS, Entebbe, Uganda; 3 African Population and Health Research Center, Nairobi, Kenya; 4 Department of Health Sciences, University of York, York, United Kingdom; 5 Department of Clinical Science and Education, Karolinska Institutet, Stockholm, Sweden; University of North Carolina at Chapel Hill, UNITED STATES

## Abstract

**Introduction:**

Homebirths are common in low and middle income countries and are associated with poor child survival. We assessed the feasibility of using smartphones by village health workers for pregnancy registration and the effectiveness of health text messages (SMS) sent to pregnant women through village health workers in reducing homebirths in rural Uganda.

**Methods:**

A non-randomised intervention study was undertaken in 26 villages. In the intervention arm, village health workers registered pregnant women (n = 262) in 13 villages using a smartphone app (doForm) and paper forms and gestation age-timed SMS were sent through village health workers to the pregnant women. In 13 control villages, (n = 263) pregnant women were registered on paper forms only and no SMS was sent. The main outcome was place of birth measured through a self-report. Logistic regression with generalised estimating equations was used to explore the effect of the intervention.

**Results:**

Comparing 795 corresponding data fields on phone and paper revealed that numeric variable fields were 86%-95% similar while text fields were 38%-48% similar. Of the 525 pregnant women followed, 83 (15.8%) delivered at home. In the adjusted analysis, the intervention was associated with lower odds of homebirths [AOR = 0.38, 95%CI (0.15–0.97)]. Muslim religion [AOR = 4.0, 95%CI (1.72–9.34)], primary or no maternal education [AOR = 2.51, 95%CI (1.00–6.35)] and health facility distance ≥ 2 km [AOR = 2.26, 95%CI (0.95–5.40)] were independently associated with homebirths.

**Conclusion:**

Village health workers can register pregnant women at home using phones and relay gestation age specific SMS to them to effectively reduce homebirths.

## Introduction

Although a substantial decline in child mortality has been observed over the past decades, the burden of neonatal mortality remains high. Sub Saharan Africa contributes half of under-five mortality globally and 44% of the deaths occur in the neonatal period [1]. Increasing access to skilled birth attendance (SBA) is among key strategies recommended by the World Health Organization (WHO) to reduce maternal and neonatal mortality [2]. Despite several efforts geared towards scaling up SBA, many women in low income countries deliver at home. A recent estimate showed that between 2011 and 2015, approximately 50% of births in sub Saharan Africa occurred at home [3]. Among several barriers to health facility delivery, lack of consistent communication on the importance of delivering at a health facility has been cited [4].

According to the 2011 Uganda Demographic and Health Survey report, only 58% of women in Uganda delivered under skilled supervision [5]. Uganda’s high maternal mortality ratio of 438/100,000 and infant mortality of 54 per 1,000 live births [5] is partly due to limited access to supervised deliveries [6]. To bridge the gap between the health system and the communities, community health workers commonly referred to as village health teams (VHTs) in Uganda were introduced by the ministry of health to promote health services including mobilisation of women to attend antenatal clinics and deliver at health facilities [7]. However, VHTs have limited education and training and may not provide accurate and consistent health information that could motivate women to take up services. The increase in mobile phone ownership in rural places presents a formidable opportunity to reinforce village health workers’ communication with pregnant women. Rapid changes in technology and falling prices make smartphones more accessible for data collection and disseminating health information in low resource settings. Although there is a growing body of literature on the promise of mobile phones for communicating health messages, most studies have tended to be small–scale donor–funded initiatives without adequate evidence base. Examples of such exploratory pilot projects in Rwanda [8], Ethiopia [9], Ghana [10], and Uganda [11] have shown that village health workers can successfully be engaged to deliver text messages. A cluster randomised trial in Zanzibar in which text messages were delivered directly to pregnant women recruited from antenatal clinics, increased facility births from 47% to 60% but only benefited women in urban areas who owned phones [12]. Women from rural communities who most needed the intervention did not benefit. We have previously shown in our study area in rural Uganda that one third of pregnant women owned phones and 11% had access to a phone of a family member or neighbour leaving 60% of rural pregnant women with no access to a phone [13]. In another project in northwestern Uganda referred to as “Text to Change” (TTC), SMS uptake was very low in vulnerable communities because they had no personal phones and a vast majority could not read text messages (SMS) [14]. Delivering SMS through VHTs may therefore present an opportunity to reach women in rural communities with accurate health information.

In this study we hypothesised that village health workers can be trained to use smartphones for pregnancy registration in rural communities and deliver gestation age specific health messages to pregnant women to improve uptake of facility births. We implemented a community based pragmatic intervention assess the feasibility of using smartphones for pregnancy registration and relaying standard health text messages to pregnant women via village health workers and evaluated the effect of the intervention on reduction of home deliveries. The ultimate goal of this intervention was to contribute information to future trials for improvement of child survival in the critical period surrounding birth.

## Methods

### Design

This was a non-randomised intervention study nested within a general population cohort (GPC) in rural south-western Uganda. Study participants and investigators were not masked due to the nature of the intervention requiring overt participation. The study was conducted from April 2014 to November 2015.

### Study setting and population

The GPC is located in Kalungu, a rural district approximately 120 kilometres west of Kampala (the capital of Uganda). The cohort, consisting of approximately 22,000 residents who are predominantly subsistence farmers was established in 1989 by the UK Medical Research Council (MRC) in collaboration with the Uganda Virus Research Institute (UVRI) to investigate HIV trends and population level risk factors. The research objectives have now been expanded to maternal and child health [15]. Twenty six villages were mapped and a census undertaken annually to document changes in households. Since 2002, pregnancy registration by village health workers using paper forms was introduced to support the prevention of mother to child transmission of HIV (PMTCT) program in the community. The study area is served by five primary health care facilities. One higher level centre within the study area and a hospital 20 km away from the study offered emergency obstetric services including caesarean sections. Between 2002 and 2012, home deliveries in the area had reduced from 46% to 37% [16]. The choice of the cohort for the intervention was a pragmatic decision based largely on study resources.

### Inclusion criteria

To be enrolled in the study, a woman of reproductive age had to be pregnant as observed by a community health worker (no pregnancy tests done) and resident in the study area for at least 3 months or intending to live longer and willing to participate in the study.

### The intervention

We introduced the intervention within one half of the study area (13 adjacent villages). The other half (13 villages) were considered as control arm. There was no formal randomization done because we intended to keep adjacent villages together to allow sharing of resources (mobile phones) among village health workers in the intervention arm and minimise contamination of study arms since the study was confined within a defined geographical area where a general population based cohort had been established.

The main objective of the intervention was to link pregnant women to their nearest primary health care facility during childbirth. The intervention had two components: registering pregnant women by village health workers using smartphones (from April 2014) and tracking pregnant women with automated SMS sent from a centralised database (from January 2015). One village health worker from each village was trained to register and follow pregnant women in their own community, filling in an electronic form customized for this purpose. We used the doForm web based application (doForms, Atlanta) [17] to construct pregnancy registration forms and then dispatched the forms to three smartphones provided to the village health workers. One smartphone was shared among four to five village health workers in adjacent villages. After filling the forms the village health workers sent back the encrypted data via the cloud to our database every month. From the database, health messages adapted from the Mobile Alliance for Maternal Action (MAMA) website [18], were automated and sent to the village health workers who in turn delivered the messages to the pregnant women based on their gestation age. The MAMA messages were derived from WHO and UNICEF guidelines developed in close collaboration with a group of global health experts to cover 5 to 42 of weeks of pregnancy. They provide information on antenatal care, safe delivery, nutrition and motivate mothers to get the right care at the right time. Each month, a health text message translated to the local language (Luganda) specifying the pregnant woman to be visited was sent from a central web database to the personal mobile phones of the village health workers. The village health workers in turn visited the pregnant women in their homes and read the messages verbatim. During the same visit, the village health worker updated the pregnancy status of the mother as; “baby delivered” or “still pregnant” or “pregnancy lost” or “mother not available at home”. The forms also captured basic demographic information.

In the control arm, village health workers registered and followed pregnancies monthly using paper registers only and did not receive any health text message. The village health workers from all study villages attended quarterly meetings during which refresher trainings were conducted. The authors (GA and LK) provided technical support to the village health workers in the field when needed.

### Sample size considerations

No formal sample size calculation was undertaken since this was a non-randomised feasibility study. The sample size of 525 was accrued on a pragmatic decision informed by our study resources to conduct the study within the 26 adjacent villages of the GPC.

### Outcome measures

The study outcome was place of delivery measured through a self-report by women to an independent team of trained interviewers who regularly conducted census within the GPC. The outcome was measured during a census conducted from January to November 2015 during which all women of reproductive age were asked if they had been pregnant in the past year, what the outcome of the pregnancy was and the place where they delivered. They were also asked if they had received a message from a village health worker and to specify what the message was. Detailed demographic information on age, tribe, education, marital status of mother and household socioeconomic status including cash and non- cash income, and Geographical Information System (GIS) data for the households and neighbouring health facilities was also collected.

### Statistical analysis

All statistical analyses were conducted using SAS 9.4 (SAS Institute Inc., Cary, NC, USA). We first compared smartphone data with paper data using the “PROC COMPARE” command which determined whether matching observations in the two dataset were comparable. Our primary outcome was place of delivery. We first computed frequencies of birth by place and month of delivery stratified by study arm to show how the incidence of home births changed over time in the intervention and control villages. Logistic regression along with generalised estimating equations to account for cluster correlation within households was used on complete data to explore the effect of the intervention on the outcome. An exchangeable working correlation was used to allow for within cluster correlation and standard errors were based on the robust covariance matrix. We estimated intracluster correlation for the outcome. Explanatory variables in Table 1 were entered in a stepwise manner and those with likelihood ratio test p<0.10 were retained resulting in a final model with age, education, tribe, religion, distance to nearest health centre, visit by village health worker, and intervention arm. Results were expressed as odds ratios (ORs) for home delivery, with 95% confidence intervals (95% CIs). Model fitness was assessed using Quasi-likelihood Information Criteria (QIC). We also estimated mortality among infants who were delivered during the SMS intervention and compared mortality in intervention and control villages.

**Table 1:** Comparison of smart phone and paper based pregnancy registration by community health workers in rural Uganda (2014–2015).

Variable	Variable type	*Same value (%)	Mean (paper)	Mean (phone)	Difference (%)
**ALL fields**		577/795 (72.6%)			
**Household number**	numeric	151/158 (95.0%)	476.70	463.50	-2.50%
**Date of registration (in SAS)**	numeric	137/158 (86.2%)	20120.00	20124.00	0.02%
**Gestation age (months)**	numeric	151/158 (95.0%)	6.15	6.11	-0.61%
**Facility birth (Yes/No)**	Character	61/158 (38.4%)	N/A	N/A	N/A
**Health facility Name**	Character	77/158 (48.4%)	N/A	N/A	N/A

*Proportion of variable values that are the same in both paper based and phone based pregnancy register

### Ethics

The study was approved by the Uganda Virus Research Institute Research Ethics Committee (REC) (GC/127/15/01/62) and the Uganda National Council for Science and Technology (HS870). Since pregnancy registration was conducted by lay community health volunteers in a population with low literacy levels and the study procedures presented no more than minimal risk to the participants, REC approved a verbal consent. The community health volunteer checked a box to record to indicate if the participant declined or accepted to participate in the study. Only those who accepted to participate were enrolled in the study.

## Results

### Registration of pregnant women and follow-up

**Fig 1:**
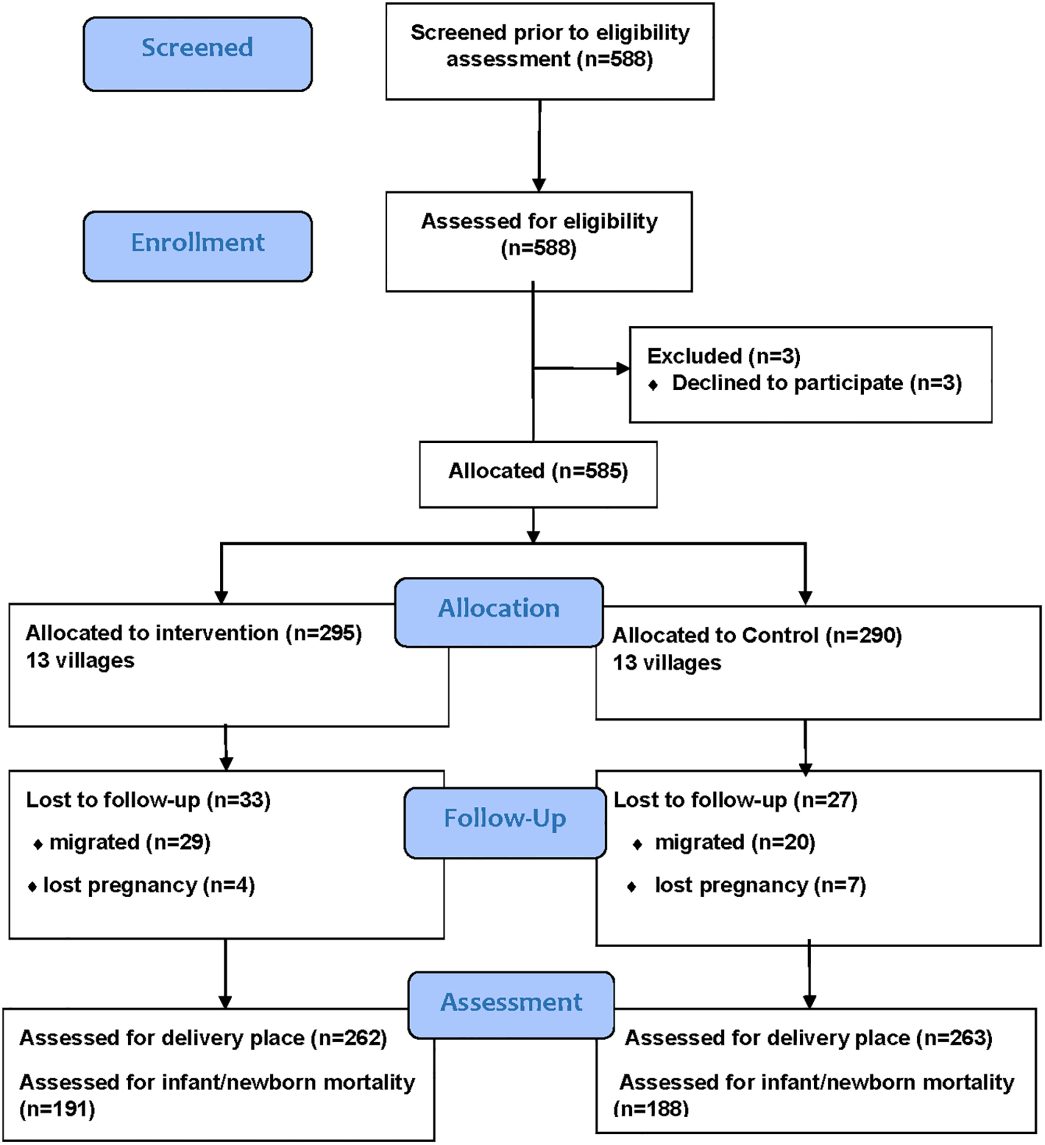
Flow chart showing enrolment and follow-up of pregnant women in rural Uganda.

In total, 588 pregnant women were approached between April 2014 and November 2015 by village health workers in the 26 villages. Of the women approached, 3 refused to participate, thus 585 (295 in the intervention and 290 in control villages) were available for follow-up. Sixty women were lost to follow-up mainly due to migration outside study area (29 in intervention and 20 in control) and pregnancy loss (4 in intervention and 7 in control) resulting in 262 women in the intervention villages and 263 in the control villages with a final outcome (Fig 1). Only one woman from each study arm crossed to the other arm during follow-up.

In the intervention arm, 234 pregnant women were registered by phone, 58 of whom were not resident. Of the 176 resident women registered by phone 158 (89.8%) were also registered using paper forms. Comparing 795 matching data entry fields showed that 72.5% of the records were exactly similar on phone and paper registers. Village health workers were better at recording numeric variables on phone than text variables. For example, 95.0% entered household numbers on phone exactly as on paper and 86.2% entered exactly the same date on phone as in paper while entering character variables yielded a lower consistency between phone and paper; 38.4% and 48.4% respectively for a “yes” or “no” response for home birth and name of health facility (Table 1). During follow-up, 437 visits were registered on phone with 97% of the women visited at least more than once (range 1–4 visits).

### Baseline characteristics of pregnant women in the study

Table 2 shows the characteristics of the 525 women who completed follow-up. In both study arms, over 80% of the pregnant women were below the age of 35 years with at a mean gestation age of 6.3 (standard deviation 1.4) months and from the main ethnic group (Buganda), one third were Muslims and about 80% were married. More women in the intervention village had attained post primary education than those in the control villages (47.5% versus 28.6%) and also lived within a distance of two kilometres from a health facility (61.0% versus 27.8%). Slightly more women in the intervention villages lived in households with a cash income equivalent to at least two dollars per day. Although the text messages were sent only to the women in the intervention villages through the village health workers, up to 40.6% of the women in the control villages reported having received a message through a village health worker.

**Table 2:** Baseline characteristics of pregnant women enrolled and followed by study arm.

Characteristics	Intervention (N = 262)		Control (N = 263)	
	n	%	n	%
**Age group (years)**				
15–24	113	43.3	107	40.8
25–34	110	42.2	115	43.9
>35	38	14.6	40	15.3
**Tribe**				
Muganda	205	80.4	187	73.9
Other	50	19.6	66	26.1
**Religion**				
Roman Catholic	154	67.8	136	60.4
Muslim	62	27.3	71	31.6
Other	11	4.9	18	8
**Education level**				
None or primary	135	52.5	180	71.4
Post primary	122	47.5	72	28.6
**Marital status**				
Single	36	14.1	34	13.3
Married	204	80	195	76.5
Divorced/Separated	15	5.9	26	10.2
**Distance from nearest Health Centre**				
< 2km	150	61	69	27.8
> = 2km	96	39	179	72.2
**Household cash income**				
<2 USD per day	94	55	102	62.2
> = 2 USD per day	77	45	62	37.8
**Was visited by community village health worker**				
Yes	152	59.4	109	41.6
No	104	40.6	153	58.4
**Received text message from village health worker**				
No	104	41.6	151	59.5
Yes	146	58.4	103	40.6
**Which message was delivered by village health worker**				
No message	3	2	4	3.7
Attend antenatal clinic	43	28.9	40	37.4
Deliver in health facility	103	69.1	63	58.9
**Calendar period of birth**				
Apr-Dec 2014	118	48	82	39
Jan- Nov 2015	128	*52*	128	61

### Place of delivery

**Fig 2:**
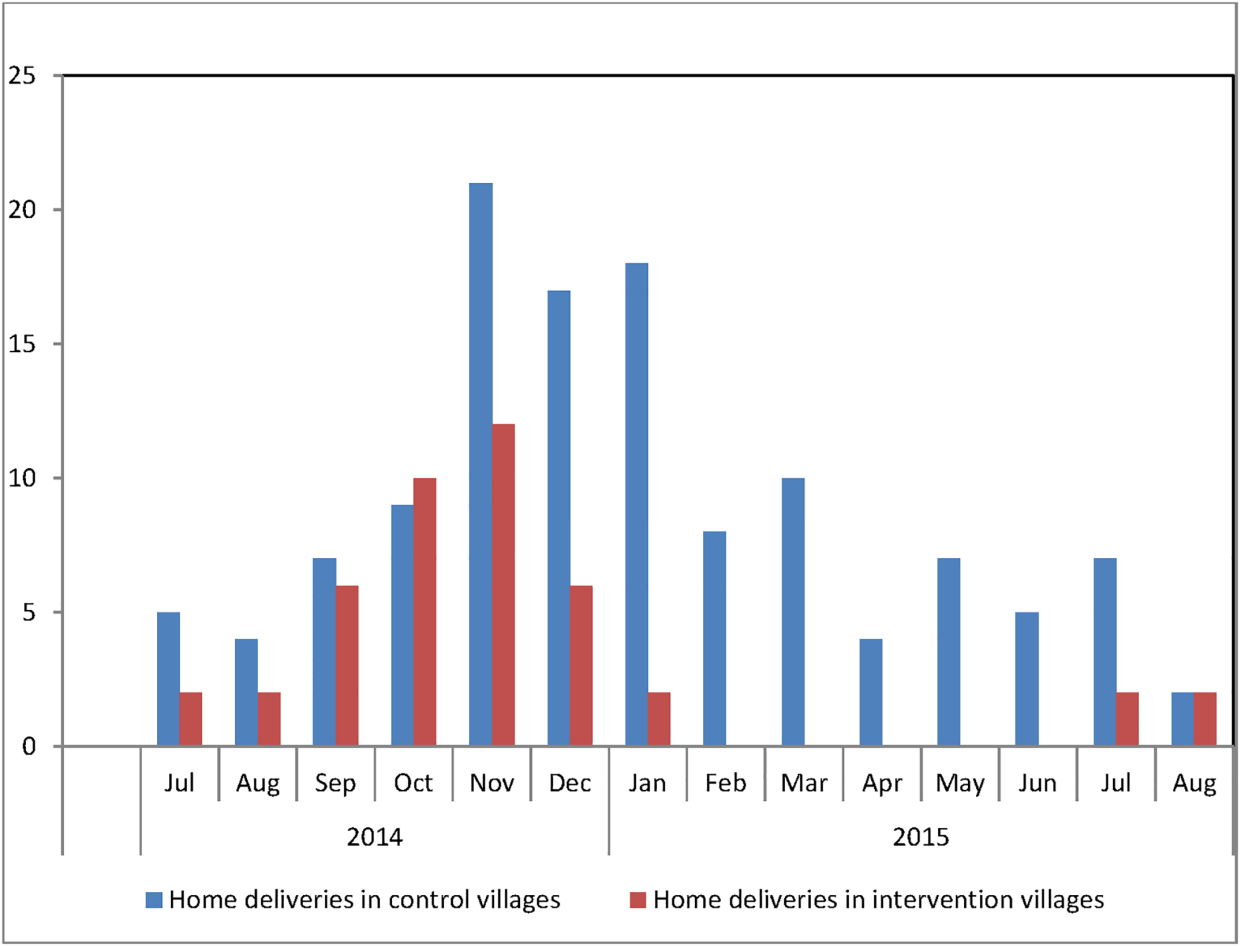
Number of home deliveries in control and intervention villages in rural Uganda (2014–2015).

Of the 525 pregnant women, 442 (84.2%) reported delivering in a health facility, 18 (3.4%) delivered at home under traditional birth attendant supervision, 54 (10.3%) were helped by a relative or a friend at home and 11 (2.1%) delivered alone. Fig 2 represents the change in incidence of deliveries in control and intervention villages and reveals substantial reduction in home deliveries after introduction of SMS in January 2015, with some months having no home births in the intervention villages. The intra-cluster correlation coefficient for the outcome was computed to be 0.25.

### Factors associated with home deliveries

Table 3 shows characteristics of women that influenced the choice of delivery place. Over all, there were fewer homebirths among women in the intervention compared to those in control villages (9.2% versus 22.4%). A higher proportion of homebirths was observed among older women (>35 years), women from non-indigenous ethnic groups, women with primary or no education, Muslims and those living two or more kilometres away from a health facility.

**Table 3:** Factors associated with home deliveries among pregnant women in rural south-western uganda (2014–2015).

	Home delivery N (%)	Unadjusted OR (95%CI)	Adjusted OR (95%CI)	p-value
**Study villages**				
Control arm	59 (22.4)	1	1	
Intervention arm	24 (9.2)	0.35 (0.21–0.58)	0.38 (0.15–0.97)	0.04
**Age group (years)**				
15–24	34 (15.5)	1	1	
25–34	29 (12.9)	0.81 (0.47–1.38)	0.87 (0.25–2.99)	0.84
>35	20 (25.6)	1.89 (1.01–3.53)	0.46 (0.19–1.09)	0.08
**Tribe**				
Muganda	57 (14.5)	1	1	
Other	26 (22.4)	1.70 (1.01–2.85)	2.07 (0.78–5.48)	0.14
**Religion**				
Roman catholic	38 (13.1)	1	1	
Muslim	28 (21.1)	1.77 (1.03–3.03)	4.00 (1.72–9.34)	<0.01
Other	5 (17.2)	1.38 (0.50–3.84)	0.23 (0.03–1.99)	0.18
**Education level**				
Post primary	16 (8.3)	1	1	
None or primary	67 (21.3)	3.01 (1.69–5.36)	2.51 (1.00–6.35)	0.05
**Distance from nearest health centre**				
< 2km	19 (8.7)	1	1	
≥ 2km	60 (21.8)	2.94 (1.69–5.10)	2.26 (0.95–5.40)	0.07
**Received text message**				
No	32 (12.9)	1	1	
Yes	49 (19.2)	0.62 (0.38–1.01)	0.50 (0.22–1.12)	0.09
**Calendar period of delivery**				
Apr-Dec 2014	25 (12.5)	1	1	
Jan-Nov 2015	45 (17.6)	1.49 (0.88–2.53)	0.75 (0.33–1.77)	0.52

In the bivariate analysis, homebirths were associated with age more than 35 years (OR = 1.89, 95%CI [1.01–3.53]), belonging to a tribe other than Buganda tribe (OR = 1.70, 95%CI [1.01–2.85]), Muslim religion (OR = 1.77, 95CI [1.03–3.03]), primary or no education (OR = 3.01, 95CI [1.69–5.36]), living two or more kilometres away from a health centre (OR = 2.94, 95%CI [1.69–5.10]), and not receiving a health text message from a community health worker (OR = 1.61, 95CI [0.99–2.62]). After adjusting for correlations within households and village, controlling for age, education level, tribe, distance from a health facility and the calendar period when birth occurred, the intervention was associated with lower odds of home delivery (AOR = 0.38, 95%CI [0.15–0.97]). Muslim religion (AOR = 4.0, 95%CI [1.72–9.34]) and primary or no maternal education (AOR = 2.51, 95%CI [1.00–6.35]) were independently associated with homebirths. Women who were more than 2 km from a health facility were also more likely to deliver from home (AOR = 2.26 [0.95–5.40]) compared to those living nearer the health facilities. Receiving a text message from a village health worker (AOR = 0.50, 95%CI [0.22–1.12]) and a woman’s age>35 years (AOR = 0.46 95%CI [0.19–1.09]) were associated with lower odds of home delivery, though with borderline statistical significance. Women who delivered between January and November 2015, during the SMS component of the intervention were also less likely to deliver at home (AOR = 0.75 95%CI (0.33–1.77), though not statistically significant.

### Comparison of mortality in the intervention and control villages

During the SMS intervention, 379 births were reported between January 2015 and November 2015, and 28 deaths occurred; 12 /191 (63 per 1000 live births) in the intervention villages compared to 16/188 (85 per 1000) in the control villages (OR = 0.72, 95%CI [0.33–1.57]). Ten of the 28 babies were born alive but died on the same day of birth; 5/191 (26 per 1000 livebirths) in the intervention compared to 5/188 (27 per 1000 live births) in the control villages (OR = 0.98, 95%CI [0.28–3.46]).

## Discussion

Our study has shown that village health workers can be effectively engaged to register pregnant women and follow them with standardised text messages in their homes. Village health workers were able to deliver the messages to 60% of pregnant women registered in the study and this was associated with a substantial reduction in home births. A cluster randomized trial in Zanzibar showed approximately 20% increase in uptake of facility deliveries among pregnant mothers attending antenatal care when the messages were directly delivered to the mothers [12]. Unlike in the Zanzibar trial, we recruited pregnant women from their homes and sent text messages through village health workers to reach women who do not own phones and are most vulnerable in rural communities. Our findings contribute to dearth of literature on mobile phone use for data collection and dissemination of specific health messages to improve maternal and child health. A recent systematic review on mHealth interventions for maternal, new-born and child health in low–and middle–income countries highlighted the scarcity of literature and lack of robust evidence [19]. Strengthening community health systems within which homebirths occur through prospective village-based registration of pregnant women has been shown to reduce neonatal mortality in India [20] and Bangladesh [21]. Pregnancy registration enables accurate estimate of population denominators that improves the targeting of services. In our study area, pregnancy registration was enhanced during the intervention and this directly contributed to the village health workers focusing their mobilisation activities to a targeted audience. The pregnant women most likely attached more value to gestation age-timed messages than general messages, thus the higher uptake of services. Although our study showed a lower infant mortality in the intervention arm, there was no difference in neonatal mortality on the first day of delivery possibly because of delayed care-seeking at the time of birth and the inability of health centres to handle new born resuscitation.

Our study was able to specifically demonstrate the ability of the village health workers to use the smartphone App (doForm). The use of doForm has the potential to increase efficiency in data collection since data can be accessed in real time to allow resolving immediate edits. Our community health workers were enthusiastic with the use of the phones similar to observations made in a South African study where community health workers preferred to use the android phones to paper for data collection because they felt that the system facilitated their work and made it easier [22].

There were some factors that deterred facility births among women in the study. These include, distance more than two kilometres to a health facility, Muslim religion, and low maternal education that have previously been observed [23]. In rural communities in Uganda, most pregnant women walk to health facilities and may thus find a distance more than two kilometres too far to cover during labour. Education improves comprehension of health information to take appropriate decisions for seeking health care, thus pregnant women with primary or less education may not perceive the value of delivering in a health facility.

Our study had some limitations. First, adjacent villages were grouped together for ease of implementation and to minimise contamination, but this caused an imbalance in the characteristics of the villages in intervention and control arms. For example women in intervention villages had better education and lived closer to health facilities. However, we collected data on these variables and adjusted for these factors during analysis. Our original aim was a cluster randomized trial, but due to limited resources the study was confined in an existing cohort that did not provide a sufficient sample and clusters for a cluster randomized trial. Information probably leaked through word of mouth from one village health worker to another or between women in adjacent control and intervention village through their social interactions in markets, places of worship (churches and mosques) or even during attendance of antenatal care. Still, the exposure to the health messages, including the advice to give birth in the health centre and not at home, was probably more widespread and intense in the intervention villages than in those referred to as “control”. Leakage of information in this context could also be perceived as beneficial to women who do not own phones. Thirdly, text message coverage in the intervention village was modest at 60%. This may be because only one village health worker in each village was assigned to register and follow all the pregnant women. Differences in terrain and size of village could have affected their ability to follow all pregnant women. Lastly, data on our primary outcome (place of delivery) was based on a self- report and therefore prone to recall and social desirability bias. If linkage of pregnancy registry with health facility records was possible, health facility birth records would have provided an alternative source of data to verify the self-reports.

### Public health implications

Despite these limitations our study has highlighted the role of mobile phones at population level pregnancy registration and in relaying specific health messages to rural pregnant women. We observed a substantial reduction in home deliveries as a result of this intervention. These findings suggest that village health workers can be engaged more meaningfully to reach women with specific health information to stimulate appropriate healthcare decisions. Properly tailored and targeted information alone seems to have had a substantial and quick impact on maternal behaviour in this rural setting with low level of material resources and “health literacy”, however policy decisions should prioritise women with low education, those living further than two kilometres from a health centre and Muslim women and their religious leaders.

### Recommendations

Registration of pregnancies by mobile phones could be improved further by minimising the use of open ended questions that require responses in text. When necessary, community health workers should be provided with pre-coded list of responses from which they select their responses. Other platforms such as use of freely available Apps or other ways of collecting data with non-android phones should be explored to minimise the cost of implementation. Spread of information by word of mouth from one pregnant woman to another was common and therefore offers a platform to investigate use of peer to peer approach as an additional strategy of delivering health messages through pregnant women who own phones to those who do not own phones.

Based on this feasibility study, a cluster randomized trial will require least 20 villages per arm (each with 20 pregnancies per year) assuming 12, as the harmonic mean number of pregnancies per village (since there is variation in number of pregnancies per village) at the 5% level with 80% power and a cluster coefficient of variation k = 0.3, using the formula for the number of clusters required in a cluster randomized trial for comparing proportions [24]. To avoid contamination the 40 villages will have to be distant from each other, randomly allocated and stratified by village size and distance from health facility.

## Supporting information

S1 Table Raw data and data dictionary for selected variables used in the analysis. (RAR)

S2 Table Consort checklist for feasibility studies. (DOC)
